# Evaluation of epigenetic methylation biomarkers for the detection of colorectal cancer using droplet digital PCR

**DOI:** 10.1038/s41598-023-35631-5

**Published:** 2023-06-01

**Authors:** J. Petit, G. Carroll, J. Zhao, E. Roper, P. Pockney, R. J. Scott

**Affiliations:** 1 Division of Surgery, John Hunter Hospital, New Lambton Heights, NSW Australia; 2 School of Medicine and Public Health, University of Newcastle, Callaghan, NSW Australia; 3 Hunter Medical Research Institute, Newcastle, Australia; 4 School of Biomedical Sciences and Pharmacy, University of Newcastle, Callaghan, NSW Australia; 5 Pathology North, Newcastle, NSW Australia

**Keywords:** Colorectal cancer, Diagnostic markers, Cancer epigenetics

## Abstract

Colorectal cancer (CRC) is the third most common cancer worldwide. Screening programs allow early diagnosis and have improved the clinical management of this disease. Aberrant DNA methylation is increasingly being explored as potential biomarkers for many types of cancers. In this study we investigate the methylation of ten target genes in 105 CRC and paired normal adjacent colonic tissue samples using a MethylLight droplet digital PCR (ML-ddPCR) assay. Receiver operator characteristic (ROC) curves were used to determine the diagnostic performance of all target genes individually and in combination. All 515 different combinations of genes showed significantly higher levels of methylation in CRC tissue. The combination of multiple target genes into a single test generally resulted in greater diagnostic accuracy when compared to single target genes. Our data confirms that ML-ddPCR is able to reliably detect significant differences in DNA methylation between CRC tissue and normal adjacent colonic tissue in a specific selection of target genes.

## Introduction

Colorectal cancer (CRC) is the third most common cancer worldwide with at least 1.9 million new cases diagnosed and over 900,000 deaths annually^1^. Screening programs for CRC vary between countries but usually involve an initial non-invasive faecal-based test. The current faecal immunochemical test (FIT) detects the presence of occult bleeding within the bowel. Participants with positive screening tests are then recommended to undergo endoscopic examination of the colon. The implementation of this process as a national screening program has been shown to reduce the risk of death from colorectal cancer as well as reducing the stage of cancer when a person is diagnosed^2,3^. Whilst this is the gold standard for diagnosis of CRC and adenomas there are limitations with this process. Firstly, colonoscopy is an invasive test and comes with potential discomfort and risk of harm to the patient. Furthermore, implementation and maintenance of a successful national screening program requires significant investment in health resources and infrastructure as well as uptake by the general population. Currently, in Australia there is only a 42% participation rate in the National Bowel Cancer Screening Program (NBCSP)^3^. This poor participation rate has been shown to be partially due to a general preference for blood-based tests rather than faecal-based tests, 78% vs 22%, respectively^4^.

The development of a highly accurate genetic blood test can potentially address both these issues. Firstly, the development of a genetic-based biomarker that is more precise than the current screening test could reduce the number of negative colonoscopies, defined as: screening colonoscopies that are performed and find no pathology. This is a necessary consequence of a colorectal cancer screening program but by improving the accuracy and precision of the test the overall number of these can be reduced. Thus, the healthcare cost and overall risk of complications for patients would both be reduced. Furthermore, a blood-based test has the potential to increase the participation rate in the NBCSP and simultaneously improve the ease at which General Practitioner led screening can be achieved through inclusion of the test in routine bloods performed for appropriately selected patients.

New genetic screening tests are beginning to emerge for CRC and one of the main areas of focus in this field is identifying tumour-specific methylome patterns^5^. Aberrant epigenetic methylation patterns are associated with many types of cancers and are considered one of the key mechanisms of tumour suppressor gene inactivation that ultimately contributes to carcinogenesis^6,7^. Hypermethylation of CpG islands within the promoter region of genes is a normal regulatory cell function that leads to silencing of transcription. However, when this normal process is disturbed and results in transcriptional silencing of tumour suppressor genes then the cells gain a growth advantage similar to that observed in classical mutation acquired cancers. Numerous hypermethylated genes have been studied in CRC and as a result, there are new methylated epigenetic biomarkers that are beginning to emerge which potentially offer a higher level of precision when compared to current tests^8–10^. This is because they are based upon individual cancer genetics rather than detection of non-specific bleeding from the colon. However, to date the clinical performance of these CRC biomarkers are not suitable for screening or initial diagnostic purposes, only for monitoring of disease recurrence or response to chemotherapy treatments.

High cost and low through-put methods of genetic-based tests have resulted in poor cost-efficiency when compared to the FIT test. ML-ddPCR offers the potential to overcome some of the limitations of previous tests. The system is automated and can provide a high-throughput methodology that is highly reliable and reproducible without the need for serial dilution calibration^11^. Furthermore, ML-ddPCR is 25-fold more sensitive when compared to conventional ML-PCR which is critically important in assessing the inherently small amounts of circulating tumour DNA (ctDNA) obtained from blood samples^12^. The current study is aimed at providing the first step in validating the diagnostic potential of ten different methylated genes, using ML-ddPCR for detection, in a large cohort of colorectal cancer and normal adjacent colonic tissue samples.

## Methods

### Clinical specimens and ethics

Fresh frozen tissue from primary tumours and paired normal adjacent tissue (NAT) from CRC patients were collected from patients undergoing resection for CRC, from 2011 to 2013, at John Hunter Hospital and Newcastle Private Hospital. A total of 105 matched tumour and NAT samples were obtained during this period and used in this study. After surgical resection and macroscopic histopathological examination samples were immediately archived and stored at − 80 °C until use (range of 4–7 years storage). Complete histopathological examination and status of the tumour was confirmed by a certified pathologist and staged using the TNM system defined by the Union for International Cancer Control (UICC)^13^. Clinicopathological characteristics of the patients from whom the samples were collected are listed in Table 1. The study was conducted in accordance with the Helsinki declaration and was approved by the Hunter New England Human Research Ethics Committee (2019/ETH01147, 11/04/20/4.03). Informed consent for the collection of specimens and further genetic analysis was obtained from all patients prior to their operations.

**Table 1:** Clinicopathological features of colorectal cancer patients.

Characteristics	Number (%)
Age (median and range)	
Gender	
Male	57 (54)
Female	48 (46)
BMI (median)	27.9
CCI (median)	5
Tumour site	
Left	63 (60)
Right	39 (37)
Both	2 (2)
Unknown	1 (1)
Tumour grade	
Low/moderate	68 (65)
High	37 (35)
Tumour stage	
I	24 (23)
II	35 (33)
III	40 (38)
IV	6 (6)
Tumour	
T1	9 (9)
T2	22 (21)
T3	62 (59)
T4	11 (10)
Tx	1 (1)
Nodal status	
N0	60 (57)
N1	34 (32)
N2	10 (10)
Nx	1 (1)
LVI	
Yes	45 (43)
No	60 (57)
Metastatic disease	
Yes	6 (6)
No	99 (94)
Smoking status	
Non-smoker	65 (62)
Ex-smoker	28 (27)
Smoker	12 (11)

*BMI* body mass index, *CCI* Charlson comorbidity index.

### DNA isolation and bisulfite treatment

Genomic DNA was isolated from the fresh frozen tissue specimens using an ethanol and salt extraction method (Supplementary Material S1) and stored at − 80 °C. 500 ng–1 µg of DNA from each sample was bisulfite treated using the EZ DNA Methylation-Gold kit (Zymo Research, Irvine, Ca) according to the manufacturer’s instructions and eluted in a volume of 40 µL Elution Buffer. Unmethylated and methylated genomic DNA (Cells-to-CpG methylated and unmethylated gDNA control kit) was similarly bisulfite treated and used as positive and negative controls for PCR. The bisulfite treated DNA was then sonicated using the protocol; 15 s ON, 90 s OFF, 8 cycles, in the Bioruptor sonication device. The DNA was quantified using Qubit 2.0, ssDNA assay (Life Technologies, Carlsbad, CA) and stored at − 80 °C.

### MethylLight droplet digital PCR protocol

ML-ddPCR was performed using the Bio-Rad QX200 system. Custom primer and probe sequences were designed for the bisulfite converted methylated alleles of each gene of interest and the Actin-beta (ACTB) refence gene (Table 2, Supplementary Material S2). The 10 target genes that were chosen after systematic review of the literature have illustrated high potential as isolated colorectal cancer biomarkers^5^. Genes were chosen that were presented as having both high sensitivity and specificity for CRC, and if data was available then low methylation levels in white blood cells and a reported sensitivity of adenoma detection was preferable. A number of sequences for other genes not reported here were also tested but failed to make it passed the screening process and optimisation for ML-ddPCR. This was often due to lack of specificity for either methylated DNA or for the gene of interest. The segment of the reference gene (ACTB) that has been used has no CpG islands that would result in differentially bisulfite converted products. A second set of primers were designed for the reference gene to overcome non-specific interaction between the reference gene primers and the ITGA4 gene probe. The choice of specific target gene sequences was guided by previously identified hypermethylated regions of these genes as well as the promotor region identified using Ensembl^14^. Two different sets of primer and probe sequences were used for the IKZF1 gene. Version 1 (v1) was designed based on the CpG island and promotor region identified using Ensembl whilst Version 2 (v2) had been previously investigated^8^. Optimisation of individual assays for each gene of interest was initially performed with a temperature gradient, followed by serial dilutions of each primer and probe.

**Table 2:** Primer and probe sequences.

Target gene		Primer sequence
ACTB (a)	Forward	TGGTGATGGAGGAGGTTTAGTAAG
	Reverse	ACCAATAAAACCTACTCCTCCCTTA
	Probe	ACCACCACCCAACACACAATAACAAACA
ACTB (b)	Forward	GAGGAGGTTTAGTAAGTTTTTTGGAT
	Reverse	TACTCCTCCCTTAAAAATTACAAAAACCAC
	Probe	ACCACCACCCAACACACAATAACAAACA
BCAT1	Forward	GTTTTCGTCGCGAGAGGGTC
	Reverse	CAAAACCTAAAACAATACCCGAAACG
	Probe	FAM-CGACGAATACACGTAACGAACT-MGB
GATA5	Forward	CGAGGAAATCGCGGGGTTTTC
	Reverse	GTTACGTAACCGCACCCG
	Probe	CCATAAAAACGACCGACTCGAATCGC
IKZF1 (V1)	Forward	TGCGCGTTTCGTTTTTTGTATCG
	Reverse	GATCCCTACTCGACCTACCCCGC
	Probe	FAM-CGACCGCCTCCCGAATCGC-MGB
IKZF1 (V2)	Forward	GACGACGTATTTTTTTCGTGTTTC
	Reverse	GCGCACCTCTCGACCG
	Probe	FAM-CCCGAATCGCTACTCCGATACAAAA-MGB
IRF4	Forward	TGGGTGTTTTGGACGGTTTC
	Reverse	CGCCTACCCTCCGCG
	Probe	FAM-TCGTTTAGTTTGTGGCGATTTCGTCG- BHQ
ITGA4	Forward	TTAGCGTTTTTTGTAGTCGC
	Reverse	ACCGCTAAATAAAATCCCGAACG
	Probe	CGAAAACGCAAAACCGAACTCCGTCTCTAC
HIC1	Forward	TTCGTCGTTAGTCGGGTTC
	Reverse	AATACACCCGAAACGACCGAC
	Probe	CCGAACTATCCCGAATCCCCCGT
NPY	Forward	TCGAGGTTTTTTTTGTCGC
	Reverse	ATACTATCGAACGAACGTCT
	Probe	CGAATAAAATACAAAAAACGAATCGCGAC
SDC2	Forward	AAATTAATAAGTGAGAGGGCGTC
	Reverse	GACTCAAACTCGAAAACTCGAA
	Probe	FAM-GCGTAGGAGGAGGAAGCGAGCGTT-BHQ
SEPT9	Forward	TTTCGTCGTTGTTTTTCG
	Reverse	TCGAAATCCGAAATAATCCC
	Probe	FAM-CGTTAACCGCGAAATCCG-MGB
WIF1	Forward	CGCGTTTAGTCGTTTAAAC
	Reverse	CTCCTCGCTACCGAAA
	Probe	CGGCGTTAGGTTGCGTAGGTGCG

ML-ddPCR was performed using 1–8 µL volume (aim between 4 and 100 ng of DNA) of sample DNA in each reaction well. Stock solutions were made so that 1 µL was required in the final PCR well volume to achieve a concentration of 900 nM primers and 250 nM probes. Individual master mixes were made for all different volumes of sample used in each run. Master mixes contained 1 µL of each target and reference gene stock probe solutions, 1 µL of each target and reference gene stock primer solutions, 11 µL of ddPCR Supermix and Autoclaved Millipore water in variable volumes relative to the sample input volume. Sample and master mix were combined to achieve a total end volume in each PCR well of 22 µL. The 96-well plate was then sealed, centrifuged at 300 rpm for 5 s, gently vortexed and recentrifuged at 300 rpm. The plate-seal was removed, and the plate was then run on the QX200 AutoDG Droplet Digital PCR system, immediately foil heat sealed using the PX1 PCR Plate Sealer and run on the C1000 Touch Thermocycler. The PCR cycling conditions were 94 °C for 10 min followed by 40 cycles of 94 °C for 20 s, 52 °C for 20 s, 66 °C for 30 s and finally 98 °C for 10 min and 4 °C finishing temperature. The plate was then analysed using the QX200 Droplet Reader and QuantaSoft software (Bio-rad). The classification of droplets was made based on a pre-determined threshold for all target genes and the reference gene. However, visual inspection of all PCR wells was also performed and threshold was adjusted if the centre of the negative droplet cloud was significantly different to the rest of the PCR plate. The reliability of results using the ML-ddPCR protocol was analysed for each target gene with two separate plates using methylated control DNA (Supplementary Material S3).

### Statistical analysis

Statistical analysis was performed using SPSS. If a PCR well had < 10,000 accepted droplets, then the results were excluded and the reaction required repeating. Similarly, if there were unusual results for the droplet clouds after reading the droplets or clearly there had been an issue with the PCR reaction in an individual well then that reaction was repeated. The Methylation Index (MI) is calculated as the total methylation value of the target gene (copies/µL) divided by the total value of the reference gene (copies/µL). The target genes were analysed in isolation as well as in all two, three and four gene combinations. A total of 515 possible combinations were analysed. The combinations of genes were analysed by combining the total target gene MI values for each gene into a Cumulative Methylation Index (CMI). The optimal sensitivity and specificity of the MI and CMI for the diagnosis of CRC was determined by receiver operator characteristic (ROC) curve analysis and the Youden Index. Potential biomarker combinations were selected based on their performance using this methodology whilst maintaining a high level of sensitivity at specificities above 94%. Additionally, ROC curve and Youden Index analysis was performed for each pathological stage separately. Scatter plots and Spearman’s rank-order correlation was performed to assess the strength of relationships between two individual target genes in both the CRC tissue and NAT. Correlation was assessed as weak, moderate and strong for values 0.1–0.29, 0.3–0.49 and > 0.5, respectively. Multivariate analysis of the difference in methylation levels between normal tissue and tumour tissue was performed using the non-parametric Wilcoxon-signed rank test and the Mann–Whitney U test. Multivariate analysis using both the Mann–Whitney U test and the Kruskal Wallis H test was also performed to assess for any association between methylation levels and other potentially confounding variables such as age, gender, body mass index (BMI), co-morbidities using the Charlson Co-morbidity Index (CCI), immunosuppression, smoking status, N-stage, T-stage, size of tumour or metastatic disease.

## Results

### MI and CMI of individual target genes

**Figure 1:**
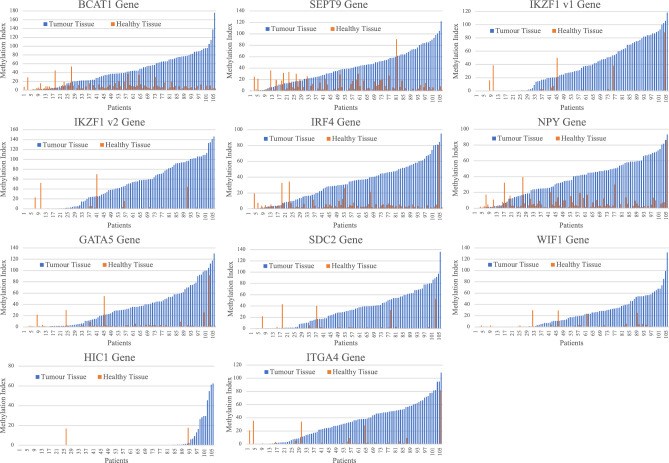
Methylation Index of tumour tissue vs healthy tissue (normal adjacent tissue) for target genes. Blue bars represent the MI of tumour tissue, orange bars represent the MI of NAT. Bars are organised from left to right in ascending value of MI according to tumour tissue. The matched NAT is adjacent to the respective tumour tissue value.

**Figure 2:**
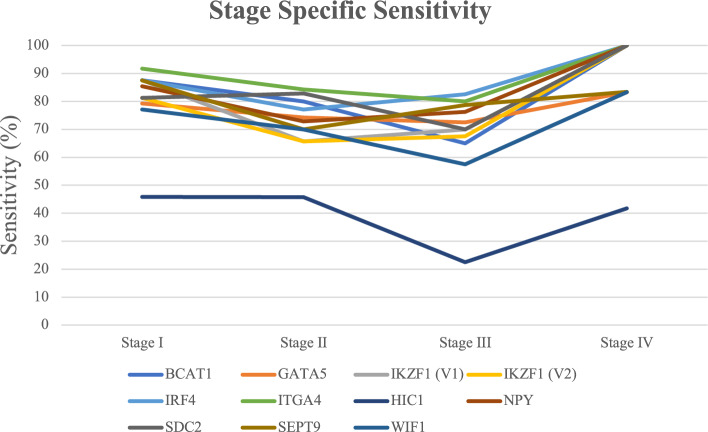
Stage specific sensitivity for individual genes.

**Figure 3:**
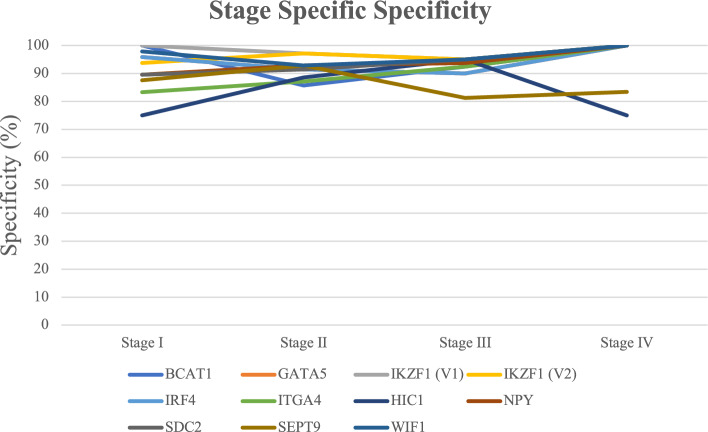
Stage specific specificity for individual genes.

The MI of the target genes all had significantly greater methylation in CRC (P < 0.0001) (Fig. 1, Supplementary Material 2). Using the ROC curves the greatest area under the curve (AUC) observed was 0.887 (*ITGA4, 95% CI 0.836–0.937*) whilst the lowest was 0.621 (HIC1, 95% CI 0.546–0.697) (Table 3, Supplementary Material S4). Except for *WIF1* and *HIC1* there was a high sensitivity and specificity found for all target genes. For each of the target genes there was a statistically significant difference in the MI between CRC tissue and NAT (Supplementary Material 2). There was a similarly high level of both sensitivity and specificity seen across all the target genes in relation to pathological stage of disease, except for *WIF1* and *HIC1* (Figs. 2 and 3, Supplementary Material S5). Notably, most of the target genes were highly methylated in early stage I and II cancers as well as later stage III and IV cancers. The CMI values showed the same characteristics as above with a statistically significantly greater methylation in CRC and a high sensitivity and specificity overall as well as in all stages of cancer (Supplementary Material 2). There were 151 combinations of target genes that had a specificity above 94% and 63 with a specificity above 95%. A total of 15 of those combinations with a specificity above 95% maintained a sensitivity above 80% (Table 4). Complete AUC, sensitivity and specificity data for all genes and combinations is listed in supplementary material S6.

**Table 3:** AUC, sensitivity and specificity for individual genes.

Gene	AUC (CI)	Sensitivity (%)	Specificity (%)	P-value
BCAT1	0.827 (0.765–0.889)	73.3–75.2	92.4–94.3	< 0.0001
GATA5	0.838 (0.78–0.895)	73.3–74.3	91.4–92.4	< 0.0001
IKZF1 (V1)	0.86 (0.805–0.914)	75.2	93.3	< 0.0001
IKZF1 (V2)	0.812 (0.749–0.875)	70.5	95.2	< 0.0001
IRF4	0.875 (0.823–0.927)	81.9–82.9	90.5–91.4	< 0.0001
ITGA4	0.887 (0.836–0.937)	82.9	88.6–89.5	< 0.0001
HIC1	0.621 (0.546–0.697)	43.8	78.1	< 0.0001
NPY	0.872 (0.819–0.924)	80	90.5	< 0.0001
SDC2	0.873 (0.823–0.923)	75.2–76.2	94.3–95.2	< 0.0001
SEPT9	0.861 (0.809–0.913)	70.5–72.4	88.6–90.5	< 0.0001
WIF1	0.749 (0.676–0.823)	65.7	96.2	< 0.0003

*AUC* Area under the curve, *CI* Confidence interval.

**Table 4:** Gene combinations with high specificity and sensitivity.

Target gene(s)	AUC	Sensitivity (%)	Specificity (%)
SEPT9/IKZF1 v2/SDC2	0.893	81.0	95.2
SEPT9/IKZF1 v2/WIF1	0.890	81.9	95.2
SEPT9/IKZF1 v1/SDC2	0.898	81.0	95.2
SEPT9/IKZF1 v1/ITGA4	0.899	80.0	95.2
SEPT9/IKZF1 v2/NPY/SDC2	0.885	81.0	95.2
SEPT9/IKZF1 v2/GATA5/SDC2	0.896	81.0	95.2
SEPT9/IKZF1 v2/SDC2/HIC1	0.892	81.0	95.2
SEPT9/IKZF1 v2/SDC2/ITGA4	0.899	81.0	95.2
SEPT9/IKZF1 v2/WIF1/HIC1	0.888	81.9	95.2
SEPT9/IKZF1 v1/IRF4/SDC2	0.894	80.0	95.2
SEPT9/IKZF1 v1/NPY/SDC2	0.889	81.0	95.2
SEPT9/IKZF1 v1/GATA5/SDC2	0.900	81.0	95.2
SEPT9/IKZF1 v1 /SDC2/HIC1	0.896	81.0	95.2
SEPT9/IKZF1 v1/SDC2/ITGA4	0.903	80.0	95.2
SEPT9/SDC2/HIC1/ITGA4	0.898	80.0	95.2

*AUC* Area under the curve.

### Intergenic correlation of MI

In the CRC tissues there was a significant and strong correlation between the majority of individual target genes in both the CRC tissue and NAT groups (p < 0.01). The two genes that exhibited a variable strength of association were *WIF1* and *HIC1* (Supplementary Material S7). WIF1 showed only a moderate association to *SEPT9* and *HIC1* but a strong correlation to all other genes. *HIC1* displayed only a weak correlation for *BCAT1*, *GATA5* and *ITGA4* (p < 0.05) and a moderate correlation with *SDC2* and WIF1 (p < 0.01). However, there was no significant correlation found between *HIC1* and *SEPT9*, *IKZF1* v1, *IKZF1* v2, *IRF4* or *NPY* (p > 0.05). The only other variation was a moderate correlation observed between SEPT9 and both *IKZF1* v2 and *GATA5* (p < 0.01). Whilst most correlations in the MI for NAT specimens were significant there was a much more variable strength of this association (Supplementary Material S8). The weakest correlation was again seen with *WIF1* and *HIC1* in the NAT samples.

### Multivariate analysis of confounding factors

In CRC tissue there was no significant association between methylation levels and gender, smoking status, immunosuppression, size of tumour, N-stage or T-stage. There was a variable association seen between the MI in CRC for age, metastatic disease and CCI. The majority of individual target genes showed no significant association to age with the exception of *BCAT1*, *GATA5*, *SDC2* and *WIF1*. A significant association to age was seen for all CMI except 22 combinations. In terms of metastatic disease there was variable level of association seen in the CMI values with a significant association seen only in the *ITGA4* and *SDC2* individual genes. There was a significant association seen between the CCI and most individual or combinations of target genes except with *HIC1*, *SEPT9*, *IKZF1* v1, *IKZF1* v2, *NPY* and 22 CMI combinations (Supplementary Material 2).

In NAT there was no significant association between methylation levels and gender, immunosuppression or metastatic disease. A significant association with age was seen for the majority of both MI and CMI except for *HIC1*, *IKZF1* v1, *IKZF1* v2, *SDC2*, *GATA5* and 45 CMI combinations. Smoking status was only found to show a significant association with *GATA5*, *SEPT9* and 8 CMI combinations. There was a statistically significant association between methylation levels and CCI for all except *IKZF1* v1, *IKZF1* v2, *WIF1*, *HIC1*, *ITGA4* and 5 other CMI combinations (Supplementary Material 2).

## Discussion

The concept of using molecular tests to detect epigenetic methylation changes in circulating cell-free DNA has gained much enthusiasm as a simple and non-invasive method for CRC and adenoma population-based screening. Our results demonstrate that ML-ddPCR can reliably detect significant differences in DNA methylation between CRC tissue and normal adjacent colonic tissue. Although, not specifically addressed in this preliminary study there is potential that these combined epigenetic DNA methylation signatures can be used to identify patients with colorectal cancer. We focused on ten highly prospective target genes and most of these genes displayed high differential methylation between CRC tissue and healthy NAT (Fig. 1). Although each of the target genes performed well individually a combined marker panel was observed to have an overall higher sensitivity and specificity. This could be due to the inherent genetic variability among colorectal cancers which means that testing for a single target gene is likely to lead to more false negative results than testing for multiple targets at once.

We found that the sensitivity and specificity of the both the target genes and combined gene panels were high in early stage I and II disease. This quality is imperative for any diagnostic test in CRC since the patient outcomes of treatment for early-stage disease are significantly better than late-stage disease. Previous studies have found a variable association between stage of CRC and levels of CpG island methylation. For instance, the same research group has found various levels of association across multiple publications investigating *BCAT1 and IKZF1*^8,15,16^. Whilst most of the research suggests an increasing level of methylation with stage the results are inconsistent. Importantly, we found that ML-ddPCR is able to detect significant differences in methylation of most genes investigated in this study in stage I and II disease. However, this does not necessarily mean that this finding on tissue samples will translate through further research into clinical utility for several reasons. Primarily, the size of the tumour is likely to be smaller in these early-stage tumours. These smaller tumours represent a lower burden of disease and there is reasonable evidence to conclude a significant correlation between this and the total ctDNA^17^. The small amount of ctDNA released from these low volume tumours most likely contributes to their high false negative rate even if they are harbouring genetic changes that would produce a positive result. Therefore, although these findings validate the methodology of ML-ddPCR and the hypermethylation of these target genes found in CRC tumour tissue further research is needed to investigate their potential as CRC biomarkers.

There are several factors other than cancer that have been shown to alter CpG island methylation patterns. Smokers has been found to have a significantly altered genome-wide methylation pattern when compared to non-smokers^18^. Furthermore, complex age-related DNA methylation changes have been shown to occur throughout life. In early life, there is methylation gain globally but this is more focussed at the CpG islands and intergenic regions. However, in later life there is overall DNA methylation loss, but the CpG islands continue to gain methylation^19^. In this study the potential association of DNA methylation in the target genes to confounding variables such as age, smoking, metastatic disease, and co-morbidities is important because of the effects this could have on the utility of these genes as biomarkers. For instance, the cut-off values for a positive result may have to be altered based on age or smoking status. Similarly, these markers may not be as accurate in the presence of significant co-morbidities. The Charlson Co-morbidity Index was utilised for analysis however the individual co-morbidities that are associated with higher methylation levels is of more importance in clinical diagnostic tests. Although there is a complex relationship between CpG island methylation and potential confounding factors this does not discount the significant differences seen in this study between CRC tissue and normal colonic tissue. Although there were significant associations found between certain individual genes and the age and CCI, this study is not designed to look at these factors specifically and there is a need for more clarification on their effect on epigenetic based biomarkers.

There has been a limited number of blood-based circulating tumour DNA assays approved for clinical use. The most notable of these are Epi proColon 2.0 which detects methylated *SEPT9* and Colvera which detects methylated *BCAT1* and *IKZF1*. Both tests have had large cohort studies performed to assess their efficacy. Epi proColon 2.0 is the most studied marker and exhibits a large variation in the sensitivity (48–95%) and specificity (80–99%) between studies^20,21^. However, this range is in part due to the variability with which the results are analysed. Colvera was found to have a lower variation in sensitivity (62–77%) and specificity (89–94%) when compared to the Epi proColon 2.0 test^8,15,22,23^. Additionally, in a direct comparison to FIT the Colvera test was found to have a comparable sensitivity with slightly better specificity. However, the sensitivity for the detection of advanced adenomas was significantly higher for FIT^15^. For these reasons the Colvera test is currently only used for monitoring for disease recurrence rather than primary diagnosis or CRC screening. Despite the approval for use of these tests in the clinical setting their role has been limited due to their high cost, limited potential benefit when compared to currently used methods of detection and poor ability to detect pre-cancerous polyps. In fact, a cost-effectiveness analysis of SEPT9 methylation concluded that FIT is less costly and more effective^24^.

There are several limitations in this pilot study. Even though it was small, the sample size was adequate as a pilot study to provide preliminary data on the methodology of detection and overall statistical efficacy. The samples used here are from tissue only and although there is sufficient evidence of plasma ctDNA detection in CRC among other studies, the ability of the biomarker panels from this study to be translated into a liquid biopsy platform remains unknown at this time. Similarly, the samples used here are potentially limited by being collected from cancer and healthy tissue in the same participants. This methodology was employed to confirm that the difference in methylation was most likely due to the disease pathology itself (ie. CRC) rather than differences between individuals baseline levels of methylation of these genes. However, for these results to be translatable it will be necessary to determine the levels of methylation in people with no evidence of disease. Furthermore, there were no pre-cancerous adenoma tissues or inflammatory bowel disease specimens used in this study and therefore we cannot predict how these markers may be altered in these types of pathology.

## Conclusion

This study investigated a panel of 10 genes that have been found to show elevated levels of DNA methylation in CRC tissue compared to paired non-neoplastic colonic tissue. Eight of these genes show sufficiently altered methylation in CRC tissues to be considered candidate biomarkers for blood-based CRC diagnostic tests. The highly sensitive and reliable ML-ddPCR technique developed here will be utilised to investigate a combined marker panel in circulating tumour DNA blood samples.

## Supplementary Information

Supplementary Information 1.

Supplementary Information 2.

Supplementary Information 3.

Supplementary Information 4.

## Data Availability

The datasets used and/or analysed during the current study available from the corresponding author on reasonable request.
