# Supplementary material for: Evaluation of epigenetic methylation biomarkers for the detection of colorectal cancer using droplet digital PCR
Source: Sci Rep. 2023 Jun 1;13:8883. doi: 10.1038/s41598-023-35631-5 (PMC10235040; doi:10.1038/s41598-023-35631-5)
Supplement: Supplementary file 1 — Supplementary Information 1. [file 41598_2023_35631_MOESM1_ESM.docx]

**Supplementary information:**

Supplementary material is all available online.

**Supplementary Material 1:**

S1: Genomic DNA extraction method summary.

S2: Reference genome locations

S3: Reliability analysis.

S4: ROC curves for MI of target genes overall and by stage

S5: Stage specific sensitivity and specificity tables for individual genes

S6: AUC, Sensitivity and Specificity for all gene combinations.

S7: Spearman’s correlation CRC

S8: Spearman’s correlation NAT

**Supplementary Material 2:**

1. MI and CMI of target genes in CRC vs NAT
2. Wilcoxon signed-rank test and Mann-Whitney-U test results.
3. Kruskal-Wallis H test data of associations.

**Supplementary Material 3:**

dMIQE Checklist
