# Supplementary material for: Evaluation of epigenetic methylation biomarkers for the detection of colorectal cancer using droplet digital PCR
Source: Sci Rep. 2023 Jun 1;13:8883. doi: 10.1038/s41598-023-35631-5 (PMC10235040; doi:10.1038/s41598-023-35631-5)

**Supplementary Material 1:**

**S1:** Genomic DNA salt extraction protocol

1. To 2mL tube for TissueLyser II, add: 1.2 nuclei lysis buffer, tissue sample 5mm stainless steel beads.
2. Homogenise on TissueLyser II for 20 seconds at 30Hz, if many samples then rotate the samples at 10 seconds to result in equal homogenisation for all samples.
3. Centrifuge the sample briefly to ensure that all the tissue sample is at the bottom of the tube.
4. Add 150uL of Proteinase K and 150uL of SDS. Mix.
5. Incubate in a shaking water bath overnight at 55^0^C
6. If sample not completely digested, then add another 100uL proteinase K and repeat incubation for 1 hour.
7. Transfer and split sample into 2 x 2mL labelled tubes.
8. Add 333uL of 6M saturated salt solution. Vortex thoroughly for >30 seconds.
9. Centrifuge for 30 minutes, 1300 rpm at 4^0^C.
10. Transfer supernatant (contains DNA) into a 15mL tube.
11. Add 5mL of cold ethanol (-20^0^C to 4^0^C). Should cause DNA to precipitate with gentle inversion. If the ethanol appears dirty, then transfer the DNA into a fresh 5mL cold ethanol in 15mL tube.
12. Sample can be left overnight at 0-4C at this stage or continue process after 2-4 hours.
13. Centrifuge at 1200rpm, room temp for 2 min to pellet the DNA, remove ethanol and transfer the pellet to a 2mL tube.
14. Add 1mL of 70% ethanol and invert to wash the DNA. Ensure the pellet is in the solution and not sitting at the bottom.
15. Centrifuge at room temp, 1200rpm, for 2 mins to pellet the DNA.
16. Remove the ethanol and repeat the 70% ethanol wash steps.
17. After removal of the ethanol, air dry the pellet in the bio-safety hood to remove any trace of ethanol.
18. Resuspend the pellet in 1 x TE, 200uL. Incubate at 55C in heating block overnight to reconstitute the DNA.
19. Check that DNA has dissolved. If sample is not clear or bubbles appear trapped in a gel-like solution, then DNA is not fully dissolved. If so, then add a further 100uL of TE and repeat incubation step. Continue this until fully dissolved.
20. Use qubit to calculate concentration of sample. May then need to perform clean and concentrate step before proceeding.

**S2: Reference Genome Locations**

| Target Gene | Amplicon Length | Genome Position (GrCh38.p13) |
| --- | --- | --- |
| ACTB (a) | 132bp | Chromosome 7: 5,532,099 – 5,532,230  Antisense strand |
| ACTB (b) | 114bp | Chromosome 7: 5,532,111 – 5,532,222  Antisense strand |
| BCAT1 | 81bp | Chromosome 12: 24,948,010 – 24,948,090  Antisense strand |
|  |  |  |
|  |  |  |
| GATA5 | 122bp | Chromosome 20: 62,476,686 – 62,476,798  Antisense strand |
| IKZF1  (V1) | 97bp | Chromosome 7: 50,304,309 – 50,304,405  Sense strand |
| IKZF1  (V2) | 95bp | Chromosome 7: 50,304,271 – 50,304,365  Sense strand |
| IRF4 | 110bp | Chromosome 6: 392,036 – 392,145  Antisense strand |
| ITGA4 | 84bp | Chromosome 2: 181,457,550 – 181,457,633  Sense strand |
| HIC1 | 124bp | Chromosome 17: 2,054,938 – 2,055,061  Sense strand |
| NPY | 110bp | Chromosome 7: 24,284,127 – 24,284,237  Sense strand |
| SDC2 | 118bp | Chromosome 8: 96,494,034 – 96,494,151  Sense strand |
| SEPT9 | 96bp | Chromosome 17: 77,373,455 – 77,373,550  Sense strand |
| WIF1 | 117bp | Chromosome 12: 65,121,204 – 65,121,320  Antisense strand |

**S3: Reliability Analysis**

Plate #1

| Gene | Mean | SD | SEM | RSD |
| --- | --- | --- | --- | --- |
| BCAT1 | 40.8 | 2.6 | 1.0 | 6.5 |
| GATA5 | 33.7 | 3.5 | 1.2 | 10.4 |
| IKZF1 v1 | 40.8 | 1.0 | 0.4 | 2.5 |
| IKZF1 v2 | 33.6 | 3.1 | 1.1 | 9.3 |
| IRF4 | 34.7 | 3.4 | 1.2 | 9.7 |
| HIC1 | 42.6 | 3.5 | 1.3 | 8.2 |
| WIF1 | 34.9 | 2.4 | 0.9 | 6.9 |
| NPY | 29.1 | 3.5 | 1.2 | 11.9 |
| SDC2 | 46.6 | 4.3 | 1.5 | 9.3 |
| SEPT9 | 46.8 | 2.1 | 0.8 | 4.5 |
| ITGA4 | 23.5 | 2.7 | 1.0 | 11.5 |

SD, standard deviation; SEM, standard error of the mean; RSD, relative standard deviation

Plate #2

| Gene | Mean | SD | SEM | RSD |
| --- | --- | --- | --- | --- |
| BCAT1 | 43.8 | 2.0 | 0.7 | 4.5 |
| GATA5 | 39.8 | 5.2 | 1.8 | 13.0 |
| IKZF1 v1 | 45.7 | 2.2 | 0.8 | 4.8 |
| IKZF1 v2 | 41.8 | 3.5 | 1.2 | 8.4 |
| IRF4 | 36.6 | 3.7 | 1.3 | 10.0 |
| HIC1 | 38.6 | 3.3 | 1.2 | 8.5 |
| WIF1 | 37.8 | 4.3 | 1.5 | 11.3 |
| NPY | 34.0 | 4.0 | 1.4 | 11.9 |
| SDC2 | 47.7 | 3.4 | 1.2 | 7.0 |
| SEPT9 | 50.5 | 2.3 | 0.8 | 4.6 |
| ITGA4 | 30.6 | 3.5 | 1.2 | 11.5 |

SD, standard deviation; SEM, standard error of the mean; RSD, relative standard deviation

**S4: ROC Curves**

ROC Curve: CRC vs NAT


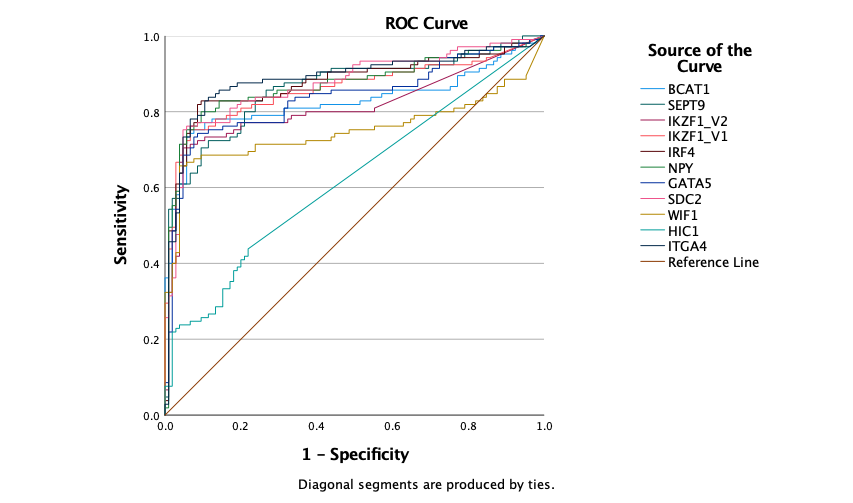


ROC Curve: Stage I vs NAT


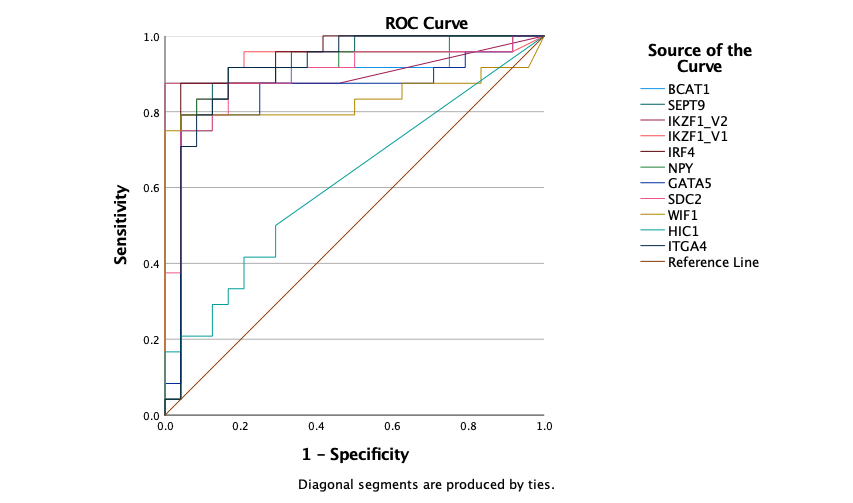


ROC Curve: Stage II vs NAT


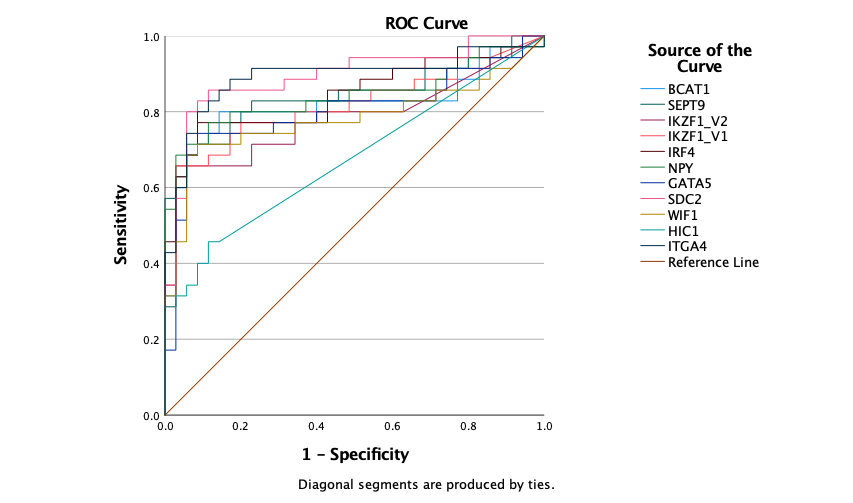


ROC Curve: Stage III vs NAT


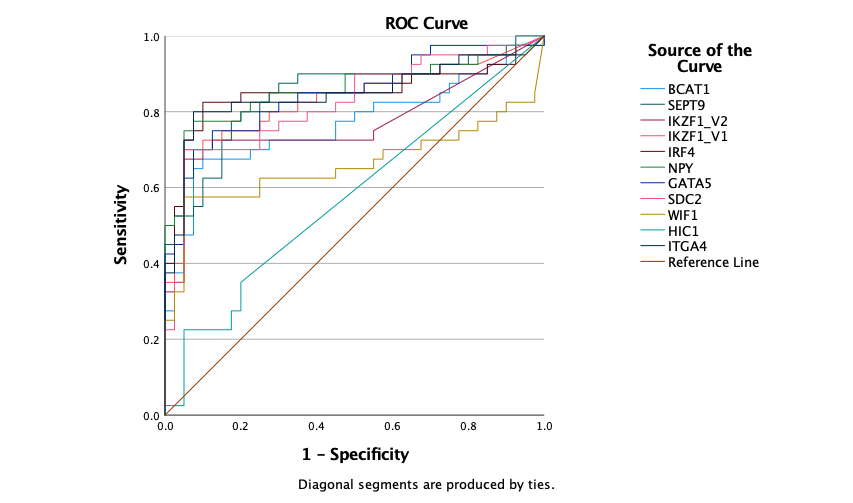


ROC Curve: Stage IV vs NAT


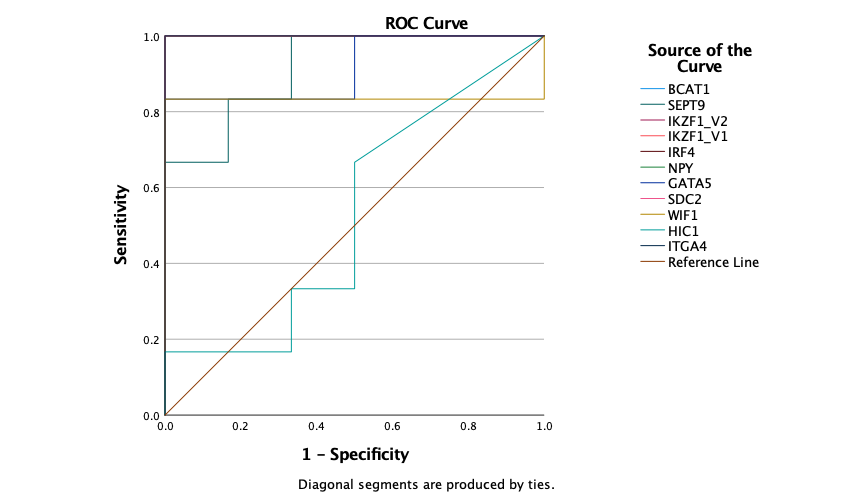


**S5:** Stage specific sensitivity and specificity tables for individual genes

S5 Table 1: Stage specific sensitivity

| Gene | Stage I (%) | Stage II (%) | Stage III (%) | Stage IV (%) |
| --- | --- | --- | --- | --- |
| BCAT1 | 87.5 | 80 | 65 | 100 |
| GATA5 | 79.2 | 74.3 | 70 – 75 | 83.3 |
| IKZF1 (V1) | 87.5 | 65.7 | 70 | 100 |
| IKZF1 (V2) | 79.2 – 83.3 | 65.7 | 67.5 | 100 |
| IRF4 | 87.5 | 77.1 | 82.5 | 100 |
| ITGA4 | 91.7 | 80 – 88.6 | 80 | 100 |
| HIC1 | 41.7 – 50 | 45.7 | 22.5 | 16.7 – 66.7 |
| NPY | 79.2 – 91.7 | 68.6 – 77.1 | 75 – 77.5 | 100 |
| SDC2 | 75 – 87.5 | 80 – 85.7 | 70 | 100 |
| SEPT9 | 87.5 | 68.6 – 71.4 | 77.5 – 80 | 66.7 – 100 |
| WIF1 | 75 – 79.2 | 68.6 – 71.4 | 57.5 | 83.3 |

S5 Table 2: Stage specific specificity

| Gene | Stage I (%) | Stage II (%) | Stage III (%) | Stage IV (%) |
| --- | --- | --- | --- | --- |
| BCAT1 | 100 | 85.7 | 92.5 | 100 |
| GATA5 | 95.8 | 91.4 | 87.5 – 92.5 | 100 |
| IKZF1 (V1) | 100 | 97.1 | 95 | 100 |
| IKZF1 (V2) | 91.7 – 95.8 | 97.1 | 95 | 100 |
| IRF4 | 95.8 | 91.4 | 90 | 100 |
| ITGA4 | 83.3 | 82.9 – 91.4 | 92.5 | 100 |
| HIC1 | 70.8 – 79.2 | 88.6 | 95 | 50 – 100 |
| NPY | 83.3 – 95.8 | 88.6 – 97.1 | 92.5 – 95 | 100 |
| SDC2 | 83.3 – 95.8 | 88.6 – 94.3 | 95 | 100 |
| SEPT9 | 87.5 | 91.4 – 94.3 | 80 – 82.5 | 66.7 – 100 |
| WIF1 | 95.8 – 100 | 91.4 – 94.3 | 95 | 100 |

**S6:** AUC, Sensitivity and Specificity for all gene combinations

| Target Gene(s) | AUC | Sensitivity | Specificity | Target Gene(s) | AUC | Sensitivity | Specificity |
| --- | --- | --- | --- | --- | --- | --- | --- |
| BCAT1 | 0.827 | 73.3 | 94.3 | **VAR0048** | 0.893 | 81.0 | 95.2 |
| GATA5 | 0.838 | 73.3 | 92.4 | **VAR0049** | 0.890 | 81.9 | 95.2 |
| OLD | 0.860 | 75.2 | 93.3 | **VAR0050** | 0.887 | 81.0 | 93.3 |
| NEW | 0.812 | 70.5 | 95.2 | **VAR0051** | 0.898 | 80.0 | 94.3 |
| IRF4 | 0.875 | 81.9 | 91.4 | **VAR0052** | 0.892 | 81.9 | 93.3 |
| ITGA4 | 0.887 | 82.9 | 89.5 | **VAR0053** | 0.886 | 81.9 | 93.3 |
| HIC1 | 0.621 | 43.8 | 78.1 | **VAR0054** | 0.898 | 81.9 | 93.3 |
| NPY | 0.872 | 80.0 | 90.5 | **VAR0055** | 0.898 | 81.0 | 95.2 |
| SDC2 | 0.873 | 75.2 | 95.2 | **VAR0056** | 0.893 | 82.9 | 94.3 |
| MS9 | 0.861 | 70.5 | 90.5 | **VAR0057** | 0.893 | 81.9 | 93.3 |
| WIF1 | 0.749 | 65.7 | 96.2 | **VAR0058** | 0.901 | 81.0 | 94.3 |
| VAR0001 | 0.875 | 81.9 | 93.3 | **VAR0059** | 0.882 | 80.0 | 93.3 |
| VAR0002 | 0.879 | 81.9 | 93.3 | **VAR0060** | 0.892 | 80.0 | 93.3 |
| VAR0003 | 0.875 | 78.1 | 93.3 | **VAR0061** | 0.891 | 80.0 | 94.3 |
| VAR0004 | 0.872 | 82.9 | 92.4 | **VAR0062** | 0.886 | 78.1 | 94.3 |
| VAR0005 | 0.876 | 79.0 | 94.3 | **VAR0063** | 0.884 | 79.0 | 92.4 |
| VAR0006 | 0.879 | 81.0 | 94.3 | **VAR0064** | 0.894 | 78.1 | 93.3 |
| VAR0007 | 0.876 | 81.9 | 92.4 | **VAR0065** | 0.886 | 81.9 | 93.3 |
| VAR0008 | 0.871 | 79.0 | 91.4 | **VAR0066** | 0.884 | 81.9 | 93.3 |
| VAR0009 | 0.879 | 79.0 | 93.3 | **VAR0067** | 0.882 | 81.9 | 94.3 |
| VAR0010 | 0.860 | 81.0 | 92.4 | **VAR0068** | 0.878 | 80.0 | 93.3 |
| VAR0011 | 0.861 | 80.0 | 92.4 | **VAR0069** | 0.887 | 81.0 | 93.3 |
| VAR0012 | 0.851 | 78.1 | 92.4 | **VAR0070** | 0.895 | 81.9 | 94.3 |
| VAR0013 | 0.856 | 77.1 | 95.2 | **VAR0071** | 0.894 | 81.9 | 93.3 |
| VAR0014 | 0.845 | 79.0 | 92.4 | **VAR0072** | 0.891 | 79.0 | 94.3 |
| VAR0015 | 0.835 | 79.0 | 92.4 | **VAR0073** | 0.901 | 79.0 | 94.3 |
| VAR0016 | 0.863 | 81.0 | 92.4 | **VAR0074** | 0.892 | 81.9 | 93.3 |
| VAR0017 | 0.867 | 81.0 | 92.4 | **VAR0075** | 0.890 | 79.0 | 95.2 |
| VAR0018 | 0.867 | 80.0 | 92.4 | **VAR0076** | 0.899 | 80.0 | 95.2 |
| VAR0019 | 0.857 | 79.0 | 91.4 | **VAR0077** | 0.883 | 78.1 | 94.3 |
| VAR0020 | 0.861 | 76.2 | 95.2 | **VAR0078** | 0.896 | 81.0 | 92.4 |
| VAR0021 | 0.851 | 80.0 | 92.4 | **VAR0079** | 0.894 | 77.1 | 95.2 |
| VAR0022 | 0.843 | 79.0 | 93.3 | **VAR0080** | 0.875 | 81.9 | 93.3 |
| VAR0023 | 0.870 | 81.0 | 92.4 | **VAR0081** | 0.889 | 80.0 | 94.3 |
| VAR0024 | 0.865 | 81.0 | 92.4 | **VAR0082** | 0.885 | 84.8 | 89.5 |
| VAR0025 | 0.864 | 80.0 | 93.3 | **VAR0083** | 0.880 | 81.0 | 93.3 |
| VAR0026 | 0.865 | 81.0 | 93.3 | **VAR0084** | 0.878 | 81.0 | 93.3 |
| VAR0027 | 0.860 | 81.0 | 92.4 | **VAR0085** | 0.887 | 81.0 | 93.3 |
| VAR0028 | 0.857 | 79.0 | 92.4 | **VAR0086** | 0.884 | 78.1 | 95.2 |
| VAR0029 | 0.866 | 81.0 | 92.4 | **VAR0087** | 0.880 | 78.1 | 95.2 |
| VAR0030 | 0.866 | 81.0 | 92.4 | **VAR0088** | 0.875 | 81.0 | 93.3 |
| VAR0031 | 0.864 | 80.0 | 93.3 | **VAR0089** | 0.873 | 78.1 | 95.2 |
| VAR0032 | 0.862 | 81.9 | 92.4 | **VAR0090** | 0.883 | 78.1 | 95.2 |
| VAR0033 | 0.858 | 80.0 | 92.4 | **VAR0091** | 0.895 | 78.1 | 94.3 |
| VAR0034 | 0.867 | 81.9 | 92.4 | **VAR0092** | 0.862 | 75.2 | 94.3 |
| VAR0035 | 0.862 | 80.0 | 93.3 | **VAR0093** | 0.860 | 79.0 | 90.5 |
| VAR0036 | 0.849 | 80.0 | 91.4 | **VAR0094** | 0.899 | 85.7 | 89.5 |
| VAR0037 | 0.846 | 78.1 | 93.3 | **VAR0095** | 0.871 | 77.1 | 94.3 |
| VAR0038 | 0.868 | 79.0 | 93.3 | **VAR0096** | 0.881 | 79.0 | 93.3 |
| VAR0039 | 0.856 | 80.0 | 92.4 | **VAR0097** | 0.894 | 84.8 | 88.6 |
| VAR0040 | 0.852 | 78.1 | 93.3 | **VAR0098** | 0.820 | 76.2 | 94.3 |
| VAR0041 | 0.868 | 79.0 | 94.3 | **VAR0099** | 0.887 | 77.1 | 94.3 |
| VAR0042 | 0.837 | 79.0 | 92.4 | **VAR0100** | 0.887 | 87.6 | 84.8 |
| VAR0043 | 0.863 | 81.0 | 92.4 | **VAR0101** | 0.879 | 81.9 | 93.3 |
| VAR0044 | 0.859 | 80.0 | 92.4 | **VAR0102** | 0.894 | 82.9 | 91.4 |
| VAR0045 | 0.889 | 81.9 | 93.3 | **VAR0103** | 0.893 | 84.8 | 90.5 |
| VAR0046 | 0.882 | 81.9 | 93.3 | **VAR0104** | 0.887 | 81.0 | 93.3 |
| VAR0047 | 0.895 | 81.9 | 94.3 | **VAR0105** | 0.887 | 81.0 | 93.3 |

| Target Gene(s) | AUC | Sensitivity | Specificity | Target Gene(s) | AUC | Sensitivity | Specificity |
| --- | --- | --- | --- | --- | --- | --- | --- |
| VAR0106 | 0.892 | 81.0 | 93.3 | **VAR0164** | 0.892 | 79.0 | 92.4 |
| VAR0107 | 0.889 | 80.0 | 93.3 | **VAR0165** | 0.889 | 74.3 | 94.3 |
| VAR0108 | 0.886 | 78.1 | 95.2 | **VAR0166** | 0.886 | 80.0 | 92.4 |
| VAR0109 | 0.882 | 81.0 | 93.3 | **VAR0167** | 0.882 | 81.0 | 93.3 |
| VAR0110 | 0.880 | 81.0 | 92.4 | **VAR0168** | 0.880 | 81.9 | 93.3 |
| VAR0111 | 0.887 | 81.9 | 92.4 | **VAR0169** | 0.887 | 78.1 | 92.4 |
| VAR0112 | 0.902 | 81.0 | 91.4 | **VAR0170** | 0.902 | 82.9 | 89.5 |
| VAR0113 | 0.874 | 75.2 | 94.3 | **VAR0171** | 0.874 | 78.1 | 94.3 |
| VAR0114 | 0.882 | 80.0 | 90.5 | **VAR0172** | 0.882 | 79.0 | 95.2 |
| VAR0115 | 0.906 | 85.7 | 89.5 | **VAR0173** | 0.906 | 77.1 | 94.3 |
| VAR0116 | 0.885 | 78.1 | 94.3 | **VAR0174** | 0.885 | 72.4 | 90.5 |
| VAR0117 | 0.893 | 80.0 | 93.3 | **VAR0175** | 0.893 | 83.8 | 88.6 |
| VAR0118 | 0.902 | 84.8 | 89.5 | **VAR0176** | 0.902 | 81.0 | 93.3 |
| VAR0119 | 0.850 | 76.2 | 95.2 | **VAR0177** | 0.850 | 78.1 | 95.2 |
| VAR0120 | 0.895 | 84.8 | 88.6 | **VAR0178** | 0.895 | 79.0 | 90.5 |
| VAR0121 | 0.897 | 83.8 | 89.5 | **VAR0179** | 0.897 | 79.0 | 93.3 |
| VAR0122 | 0.880 | 81.0 | 94.3 | **VAR0180** | 0.880 | 76.2 | 94.3 |
| VAR0123 | 0.877 | 80.0 | 94.3 | **VAR0181** | 0.877 | 70.5 | 95.2 |
| VAR0124 | 0.876 | 82.9 | 93.3 | **VAR0182** | 0.876 | 77.1 | 94.3 |
| VAR0125 | 0.872 | 80.0 | 93.3 | **VAR0183** | 0.872 | 81.0 | 93.3 |
| VAR0126 | 0.880 | 81.0 | 93.3 | **VAR0184** | 0.880 | 81.0 | 92.4 |
| VAR0127 | 0.888 | 83.8 | 91.4 | **VAR0185** | 0.888 | 79.0 | 90.5 |
| VAR0128 | 0.889 | 82.9 | 91.4 | **VAR0186** | 0.889 | 80.0 | 93.3 |
| VAR0129 | 0.885 | 79.0 | 94.3 | **VAR0187** | 0.885 | 76.2 | 95.2 |
| VAR0130 | 0.890 | 79.0 | 94.3 | **VAR0188** | 0.890 | 75.2 | 93.3 |
| VAR0131 | 0.887 | 84.8 | 89.5 | **VAR0189** | 0.887 | 83.8 | 89.5 |
| VAR0132 | 0.882 | 84.8 | 90.5 | **VAR0190** | 0.882 | 80.0 | 93.3 |
| VAR0133 | 0.889 | 84.8 | 89.5 | **VAR0191** | 0.889 | 79.0 | 94.3 |
| VAR0134 | 0.876 | 81.0 | 93.3 | **VAR0192** | 0.876 | 84.8 | 90.5 |
| VAR0135 | 0.888 | 81.0 | 93.3 | **VAR0193** | 0.888 | 81.0 | 93.3 |
| VAR0136 | 0.884 | 80.0 | 93.3 | **VAR0194** | 0.884 | 82.9 | 91.4 |
| VAR0137 | 0.884 | 79.0 | 94.3 | **VAR0195** | 0.884 | 80.0 | 93.3 |
| VAR0138 | 0.885 | 81.0 | 93.3 | **VAR0196** | 0.885 | 79.0 | 94.3 |
| VAR0139 | 0.881 | 79.0 | 94.3 | **VAR0197** | 0.881 | 79.0 | 95.2 |
| VAR0140 | 0.887 | 79.0 | 94.3 | **VAR0198** | 0.887 | 80.0 | 94.3 |
| VAR0141 | 0.881 | 81.0 | 93.3 | **VAR0199** | 0.881 | 80.0 | 90.5 |
| VAR0142 | 0.875 | 79.0 | 95.2 | **VAR0200** | 0.875 | 78.1 | 95.2 |
| VAR0143 | 0.884 | 79.0 | 95.2 | **VAR0201** | 0.884 | 76.2 | 95.2 |
| VAR0144 | 0.871 | 80.0 | 94.3 | **VAR0202** | 0.871 | 73.3 | 93.3 |
| VAR0145 | 0.883 | 81.0 | 93.3 | **VAR0203** | 0.883 | 73.3 | 92.4 |
| VAR0146 | 0.879 | 81.0 | 93.3 | **VAR0204** | 0.879 | 84.8 | 89.5 |
| VAR0147 | 0.883 | 79.0 | 92.4 | **VAR0205** | 0.883 | 79.0 | 93.3 |
| VAR0148 | 0.888 | 76.2 | 95.2 | **VAR0206** | 0.888 | 75.2 | 95.2 |
| VAR0149 | 0.900 | 80.0 | 94.3 | **VAR0207** | 0.900 | 78.1 | 95.2 |
| VAR0150 | 0.842 | 73.3 | 93.3 | **VAR0208** | 0.842 | 65.7 | 96.2 |
| VAR0151 | 0.892 | 84.8 | 88.6 | **VAR0209** | 0.892 | 77.1 | 94.3 |
| VAR0152 | 0.892 | 83.8 | 90.5 | **VAR0210** | 0.892 | 83.8 | 88.6 |
| VAR0153 | 0.867 | 79.0 | 93.3 | **VAR0211** | 0.867 | 81.9 | 93.3 |
| VAR0154 | 0.894 | 85.7 | 87.6 | **VAR0212** | 0.894 | 81.9 | 93.3 |
| VAR0155 | 0.894 | 77.1 | 95.2 | **VAR0213** | 0.894 | 81.0 | 94.3 |
| VAR0156 | 0.882 | 77.1 | 94.3 | **VAR0214** | 0.882 | 81.9 | 94.3 |
| VAR0157 | 0.870 | 77.1 | 91.4 | **VAR0215** | 0.870 | 82.9 | 93.3 |
| VAR0158 | 0.837 | 79.0 | 92.4 | **VAR0216** | 0.837 | 81.9 | 93.3 |
| VAR0159 | 0.844 | 79.0 | 93.3 | **VAR0217** | 0.844 | 81.9 | 93.3 |
| VAR0160 | 0.859 | 79.0 | 92.4 | **VAR0218** | 0.859 | 81.9 | 93.3 |
| VAR0161 | 0.859 | 80.0 | 92.4 | **VAR0219** | 0.859 | 81.9 | 93.3 |
| VAR0162 | 0.847 | 78.1 | 93.3 | **VAR0220** | 0.847 | 81.9 | 93.3 |
| VAR0163 | 0.853 | 78.1 | 93.3 | **VAR0221** | 0.853 | 81.9 | 93.3 |

| Target Gene(s) | AUC | Sensitivity | Specificity | Target Gene(s) | AUC | Sensitivity | Specificity |
| --- | --- | --- | --- | --- | --- | --- | --- |
| VAR0222 | 0.880 | 82.9 | 93.3 | **VAR0280** | 0.855 | 79.0 | 91.4 |
| VAR0223 | 0.879 | 81.9 | 93.3 | **VAR0281** | 0.875 | 81.0 | 92.4 |
| VAR0224 | 0.884 | 81.9 | 93.3 | **VAR0282** | 0.862 | 79.0 | 93.3 |
| VAR0225 | 0.874 | 81.9 | 92.4 | **VAR0283** | 0.860 | 76.2 | 95.2 |
| VAR0226 | 0.879 | 80.0 | 94.3 | **VAR0284** | 0.874 | 81.0 | 92.4 |
| VAR0227 | 0.879 | 81.9 | 93.3 | **VAR0285** | 0.850 | 80.0 | 92.4 |
| VAR0228 | 0.877 | 81.9 | 92.4 | **VAR0286** | 0.870 | 81.9 | 92.4 |
| VAR0229 | 0.874 | 79.0 | 93.3 | **VAR0287** | 0.870 | 81.0 | 92.4 |
| VAR0230 | 0.881 | 81.0 | 92.4 | **VAR0288** | 0.868 | 80.0 | 93.3 |
| VAR0231 | 0.876 | 82.9 | 92.4 | **VAR0289** | 0.868 | 79.0 | 94.3 |
| VAR0232 | 0.875 | 82.9 | 92.4 | **VAR0290** | 0.867 | 81.9 | 92.4 |
| VAR0233 | 0.874 | 81.9 | 93.3 | **VAR0291** | 0.864 | 81.0 | 92.4 |
| VAR0234 | 0.871 | 82.9 | 92.4 | **VAR0292** | 0.868 | 81.9 | 92.4 |
| VAR0235 | 0.877 | 82.9 | 92.4 | **VAR0293** | 0.870 | 81.0 | 93.3 |
| VAR0236 | 0.881 | 81.9 | 94.3 | **VAR0294** | 0.866 | 81.0 | 92.4 |
| VAR0237 | 0.877 | 81.9 | 93.3 | **VAR0295** | 0.863 | 80.0 | 93.3 |
| VAR0238 | 0.876 | 79.0 | 94.3 | **VAR0296** | 0.871 | 80.0 | 93.3 |
| VAR0239 | 0.883 | 81.0 | 93.3 | **VAR0297** | 0.868 | 81.9 | 92.4 |
| VAR0240 | 0.880 | 82.9 | 93.3 | **VAR0298** | 0.864 | 81.0 | 93.3 |
| VAR0241 | 0.878 | 81.9 | 93.3 | **VAR0299** | 0.872 | 79.0 | 94.3 |
| VAR0242 | 0.883 | 81.0 | 94.3 | **VAR0300** | 0.860 | 81.0 | 92.4 |
| VAR0243 | 0.874 | 81.9 | 92.4 | **VAR0301** | 0.867 | 81.0 | 92.4 |
| VAR0244 | 0.880 | 81.9 | 93.3 | **VAR0302** | 0.865 | 81.0 | 92.4 |
| VAR0245 | 0.878 | 79.0 | 93.3 | **VAR0303** | 0.869 | 80.0 | 93.3 |
| VAR0246 | 0.865 | 81.0 | 92.4 | **VAR0304** | 0.867 | 81.9 | 92.4 |
| VAR0247 | 0.867 | 81.0 | 92.4 | **VAR0305** | 0.865 | 81.0 | 92.4 |
| VAR0248 | 0.867 | 80.0 | 94.3 | **VAR0306** | 0.870 | 81.9 | 92.4 |
| VAR0249 | 0.862 | 81.9 | 92.4 | **VAR0307** | 0.866 | 81.9 | 92.4 |
| VAR0250 | 0.860 | 81.0 | 92.4 | **VAR0308** | 0.863 | 80.0 | 93.3 |
| VAR0251 | 0.869 | 79.0 | 94.3 | **VAR0309** | 0.870 | 81.9 | 92.4 |
| VAR0252 | 0.867 | 81.0 | 92.4 | **VAR0310** | 0.861 | 81.9 | 92.4 |
| VAR0253 | 0.864 | 81.0 | 92.4 | **VAR0311** | 0.868 | 82.9 | 92.4 |
| VAR0254 | 0.862 | 81.9 | 92.4 | **VAR0312** | 0.865 | 81.9 | 92.4 |
| VAR0255 | 0.860 | 80.0 | 92.4 | **VAR0313** | 0.862 | 80.0 | 92.4 |
| VAR0256 | 0.867 | 81.9 | 92.4 | **VAR0314** | 0.860 | 80.0 | 93.3 |
| VAR0257 | 0.864 | 76.2 | 95.2 | **VAR0315** | 0.874 | 80.0 | 93.3 |
| VAR0258 | 0.853 | 77.1 | 93.3 | **VAR0316** | 0.848 | 80.0 | 91.4 |
| VAR0259 | 0.850 | 78.1 | 92.4 | **VAR0317** | 0.869 | 81.0 | 92.4 |
| VAR0260 | 0.871 | 80.0 | 92.4 | **VAR0318** | 0.867 | 79.0 | 93.3 |
| VAR0261 | 0.857 | 80.0 | 92.4 | **VAR0319** | 0.855 | 80.0 | 92.4 |
| VAR0262 | 0.855 | 77.1 | 95.2 | **VAR0320** | 0.870 | 81.9 | 92.4 |
| VAR0263 | 0.869 | 79.0 | 94.3 | **VAR0321** | 0.868 | 79.0 | 94.3 |
| VAR0264 | 0.844 | 79.0 | 92.4 | **VAR0322** | 0.862 | 81.0 | 92.4 |
| VAR0265 | 0.865 | 81.9 | 92.4 | **VAR0323** | 0.883 | 81.9 | 93.3 |
| VAR0266 | 0.862 | 81.0 | 92.4 | **VAR0324** | 0.892 | 81.0 | 94.3 |
| VAR0267 | 0.869 | 81.0 | 92.4 | **VAR0325** | 0.891 | 81.9 | 94.3 |
| VAR0268 | 0.871 | 81.0 | 92.4 | **VAR0326** | 0.887 | 82.9 | 93.3 |
| VAR0269 | 0.872 | 80.0 | 93.3 | **VAR0327** | 0.888 | 81.9 | 93.3 |
| VAR0270 | 0.867 | 81.9 | 92.4 | **VAR0328** | 0.894 | 81.9 | 93.3 |
| VAR0271 | 0.866 | 81.0 | 92.4 | **VAR0329** | 0.887 | 81.9 | 94.3 |
| VAR0272 | 0.873 | 81.0 | 92.4 | **VAR0330** | 0.885 | 81.0 | 95.2 |
| VAR0273 | 0.870 | 81.0 | 92.4 | **VAR0331** | 0.882 | 82.9 | 93.3 |
| VAR0274 | 0.870 | 81.0 | 92.4 | **VAR0332** | 0.881 | 81.9 | 93.3 |
| VAR0275 | 0.867 | 81.9 | 92.4 | **VAR0333** | 0.888 | 82.9 | 93.3 |
| VAR0276 | 0.866 | 80.0 | 92.4 | **VAR0334** | 0.896 | 81.0 | 95.2 |
| VAR0277 | 0.872 | 81.9 | 92.4 | **VAR0335** | 0.895 | 82.9 | 93.3 |
| VAR0278 | 0.869 | 76.2 | 95.2 | **VAR0336** | 0.894 | 81.9 | 94.3 |
| VAR0279 | 0.859 | 79.0 | 92.4 | **VAR0337** | 0.901 | 79.0 | 95.2 |

| Target Gene(s) | AUC | Sensitivity | Specificity | Target Gene(s) | AUC | Sensitivity | Specificity |
| --- | --- | --- | --- | --- | --- | --- | --- |
| VAR0338 | 0.893 | 82.9 | 94.3 | **VAR0396** | 0.890 | 81.9 | 93.3 |
| VAR0339 | 0.892 | 81.0 | 95.2 | **VAR0397** | 0.899 | 81.0 | 94.3 |
| VAR0340 | 0.899 | 81.0 | 95.2 | **VAR0398** | 0.898 | 80.0 | 95.2 |
| VAR0341 | 0.888 | 81.9 | 95.2 | **VAR0399** | 0.893 | 81.0 | 92.4 |
| VAR0342 | 0.896 | 81.0 | 94.3 | **VAR0400** | 0.882 | 80.0 | 94.3 |
| VAR0343 | 0.897 | 80.0 | 94.3 | **VAR0401** | 0.878 | 79.0 | 95.2 |
| VAR0344 | 0.885 | 81.9 | 93.3 | **VAR0402** | 0.877 | 81.9 | 93.3 |
| VAR0345 | 0.895 | 81.9 | 93.3 | **VAR0403** | 0.874 | 81.9 | 93.3 |
| VAR0346 | 0.894 | 80.0 | 95.2 | **VAR0404** | 0.881 | 79.0 | 95.2 |
| VAR0347 | 0.891 | 82.9 | 93.3 | **VAR0405** | 0.891 | 83.8 | 91.4 |
| VAR0348 | 0.891 | 81.9 | 93.3 | **VAR0406** | 0.890 | 82.9 | 91.4 |
| VAR0349 | 0.897 | 81.9 | 93.3 | **VAR0407** | 0.888 | 80.0 | 94.3 |
| VAR0350 | 0.889 | 81.9 | 93.3 | **VAR0408** | 0.892 | 81.0 | 92.4 |
| VAR0351 | 0.889 | 81.0 | 95.2 | **VAR0409** | 0.888 | 81.0 | 93.3 |
| VAR0352 | 0.886 | 82.9 | 93.3 | **VAR0410** | 0.884 | 78.1 | 95.2 |
| VAR0353 | 0.885 | 81.9 | 93.3 | **VAR0411** | 0.891 | 83.8 | 90.5 |
| VAR0354 | 0.890 | 82.9 | 93.3 | **VAR0412** | 0.879 | 81.0 | 93.3 |
| VAR0355 | 0.900 | 81.0 | 95.2 | **VAR0413** | 0.889 | 81.9 | 93.3 |
| VAR0356 | 0.897 | 82.9 | 93.3 | **VAR0414** | 0.886 | 81.0 | 93.3 |
| VAR0357 | 0.897 | 81.9 | 93.3 | **VAR0415** | 0.886 | 78.1 | 95.2 |
| VAR0358 | 0.903 | 81.9 | 93.3 | **VAR0416** | 0.885 | 81.0 | 93.3 |
| VAR0359 | 0.898 | 82.9 | 94.3 | **VAR0417** | 0.882 | 78.1 | 95.2 |
| VAR0360 | 0.896 | 81.0 | 95.2 | **VAR0418** | 0.889 | 78.1 | 95.2 |
| VAR0361 | 0.903 | 80.0 | 95.2 | **VAR0419** | 0.882 | 79.0 | 95.2 |
| VAR0362 | 0.892 | 82.9 | 94.3 | **VAR0420** | 0.878 | 78.1 | 95.2 |
| VAR0363 | 0.900 | 82.9 | 93.3 | **VAR0421** | 0.886 | 78.1 | 95.2 |
| VAR0364 | 0.900 | 81.0 | 94.3 | **VAR0422** | 0.874 | 81.0 | 93.3 |
| VAR0365 | 0.886 | 82.9 | 93.3 | **VAR0423** | 0.885 | 81.9 | 93.3 |
| VAR0366 | 0.886 | 81.9 | 93.3 | **VAR0424** | 0.882 | 78.1 | 95.2 |
| VAR0367 | 0.883 | 81.9 | 93.3 | **VAR0425** | 0.888 | 80.0 | 92.4 |
| VAR0368 | 0.881 | 80.0 | 93.3 | **VAR0426** | 0.893 | 78.1 | 94.3 |
| VAR0369 | 0.886 | 81.9 | 93.3 | **VAR0427** | 0.900 | 77.1 | 95.2 |
| VAR0370 | 0.894 | 81.9 | 94.3 | **VAR0428** | 0.860 | 75.2 | 94.3 |
| VAR0371 | 0.892 | 81.9 | 93.3 | **VAR0429** | 0.898 | 85.7 | 88.6 |
| VAR0372 | 0.890 | 79.0 | 94.3 | **VAR0430** | 0.898 | 85.7 | 89.5 |
| VAR0373 | 0.897 | 81.0 | 93.3 | **VAR0431** | 0.869 | 77.1 | 94.3 |
| VAR0374 | 0.891 | 82.9 | 93.3 | **VAR0432** | 0.892 | 77.1 | 95.2 |
| VAR0375 | 0.890 | 80.0 | 94.3 | **VAR0433** | 0.894 | 84.8 | 88.6 |
| VAR0376 | 0.897 | 81.9 | 93.3 | **VAR0434** | 0.885 | 77.1 | 94.3 |
| VAR0377 | 0.884 | 79.0 | 94.3 | **VAR0435** | 0.885 | 81.0 | 93.3 |
| VAR0378 | 0.894 | 81.9 | 92.4 | **VAR0436** | 0.884 | 79.0 | 95.2 |
| VAR0379 | 0.893 | 79.0 | 93.3 | **VAR0437** | 0.881 | 81.9 | 93.3 |
| VAR0380 | 0.888 | 81.9 | 94.3 | **VAR0438** | 0.878 | 81.9 | 93.3 |
| VAR0381 | 0.887 | 82.9 | 93.3 | **VAR0439** | 0.885 | 81.9 | 93.3 |
| VAR0382 | 0.885 | 81.9 | 93.3 | **VAR0440** | 0.896 | 83.8 | 91.4 |
| VAR0383 | 0.891 | 82.9 | 93.3 | **VAR0441** | 0.894 | 81.0 | 93.3 |
| VAR0384 | 0.885 | 82.9 | 93.3 | **VAR0442** | 0.892 | 82.9 | 91.4 |
| VAR0385 | 0.883 | 81.9 | 93.3 | **VAR0443** | 0.896 | 78.1 | 95.2 |
| VAR0386 | 0.890 | 82.9 | 93.3 | **VAR0444** | 0.893 | 81.0 | 93.3 |
| VAR0387 | 0.880 | 81.9 | 94.3 | **VAR0445** | 0.891 | 84.8 | 90.5 |
| VAR0388 | 0.887 | 83.8 | 92.4 | **VAR0446** | 0.895 | 84.8 | 90.5 |
| VAR0389 | 0.886 | 81.0 | 93.3 | **VAR0447** | 0.885 | 81.0 | 93.3 |
| VAR0390 | 0.896 | 82.9 | 93.3 | **VAR0448** | 0.893 | 81.9 | 93.3 |
| VAR0391 | 0.894 | 81.9 | 94.3 | **VAR0449** | 0.891 | 81.0 | 93.3 |
| VAR0392 | 0.902 | 81.9 | 94.3 | **VAR0450** | 0.890 | 78.1 | 95.2 |
| VAR0393 | 0.892 | 81.9 | 93.3 | **VAR0451** | 0.889 | 81.0 | 93.3 |
| VAR0394 | 0.900 | 81.0 | 93.3 | **VAR0452** | 0.887 | 80.0 | 93.3 |
| VAR0395 | 0.900 | 79.0 | 94.3 | **VAR0453** | 0.892 | 78.1 | 95.2 |

| Target Gene(s) | AUC | Sensitivity | Specificity | Target Gene(s) | AUC | Sensitivity | Specificity |
| --- | --- | --- | --- | --- | --- | --- | --- |
| VAR0454 | 0.888 | 81.0 | 93.3 | **VAR0480** | 0.892 | 84.8 | 91.4 |
| VAR0455 | 0.884 | 78.1 | 95.2 | **VAR0481** | 0.887 | 83.8 | 91.4 |
| VAR0456 | 0.891 | 81.9 | 92.4 | **VAR0482** | 0.893 | 83.8 | 90.5 |
| VAR0457 | 0.881 | 81.0 | 93.3 | **VAR0483** | 0.888 | 82.9 | 91.4 |
| VAR0458 | 0.889 | 81.0 | 93.3 | **VAR0484** | 0.893 | 81.0 | 93.3 |
| VAR0459 | 0.886 | 81.9 | 92.4 | **VAR0485** | 0.889 | 79.0 | 94.3 |
| VAR0460 | 0.895 | 80.0 | 92.4 | **VAR0486** | 0.885 | 84.8 | 89.5 |
| VAR0461 | 0.901 | 81.0 | 91.4 | **VAR0487** | 0.893 | 81.9 | 93.3 |
| VAR0462 | 0.906 | 85.7 | 88.6 | **VAR0488** | 0.887 | 84.8 | 89.5 |
| VAR0463 | 0.872 | 76.2 | 93.3 | **VAR0489** | 0.886 | 81.0 | 93.3 |
| VAR0464 | 0.903 | 84.8 | 88.6 | **VAR0490** | 0.888 | 80.0 | 93.3 |
| VAR0465 | 0.905 | 85.7 | 89.5 | **VAR0491** | 0.883 | 79.0 | 94.3 |
| VAR0466 | 0.883 | 78.1 | 94.3 | **VAR0492** | 0.889 | 78.1 | 95.2 |
| VAR0467 | 0.898 | 85.7 | 88.6 | **VAR0493** | 0.883 | 81.0 | 93.3 |
| VAR0468 | 0.900 | 84.8 | 89.5 | **VAR0494** | 0.889 | 81.9 | 92.4 |
| VAR0469 | 0.894 | 84.8 | 88.6 | **VAR0495** | 0.886 | 79.0 | 94.3 |
| VAR0470 | 0.882 | 81.9 | 93.3 | **VAR0496** | 0.879 | 81.0 | 93.3 |
| VAR0471 | 0.883 | 82.9 | 92.4 | **VAR0497** | 0.888 | 81.0 | 93.3 |
| VAR0472 | 0.880 | 81.0 | 94.3 | **VAR0498** | 0.883 | 79.0 | 95.2 |
| VAR0473 | 0.885 | 80.0 | 94.3 | **VAR0499** | 0.881 | 81.0 | 93.3 |
| VAR0474 | 0.881 | 81.9 | 93.3 | **VAR0500** | 0.881 | 79.0 | 92.4 |
| VAR0475 | 0.876 | 80.0 | 94.3 | **VAR0501** | 0.901 | 81.9 | 91.4 |
| VAR0476 | 0.882 | 80.0 | 94.3 | **VAR0502** | 0.900 | 80.0 | 94.3 |
| VAR0477 | 0.874 | 82.9 | 93.3 | **VAR0503** | 0.889 | 84.8 | 88.6 |
| VAR0478 | 0.883 | 82.9 | 93.3 | **VAR0504** | 0.893 | 85.7 | 87.6 |
| VAR0479 | 0.879 | 81.0 | 93.3 |  |  |  |  |

**S7: Spearman’s Correlation (CRC)**


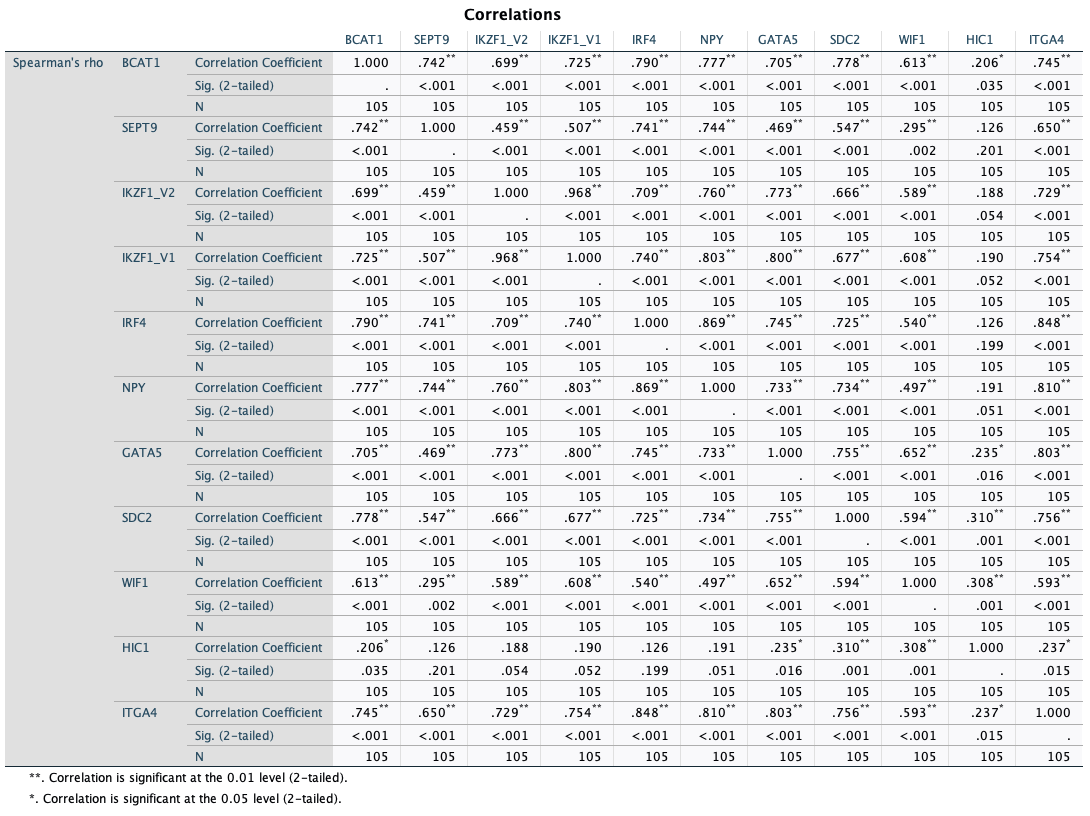


**S8: Spearman’s Correlation (NAT)**


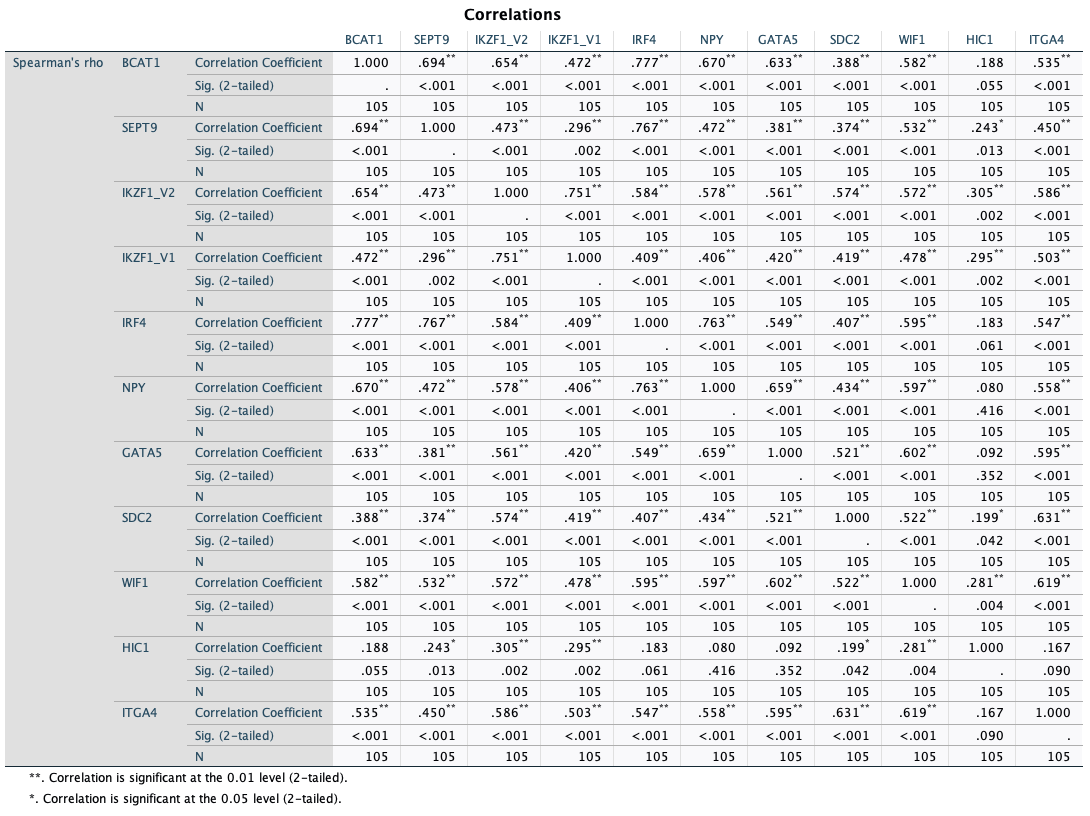

Supplement: Supplementary file 2 — Supplementary Information 2. [file 41598_2023_35631_MOESM2_ESM.docx]
